# Transferência de tecnologia para vacina contra COVID-19 no Instituto de Tecnologia em Imunobiológicos (Bio-Manguinhos), Fundação Oswaldo Cruz

**DOI:** 10.1590/0102-311XPT120023

**Published:** 2024-05-17

**Authors:** Vivianne Zalmon Rosenberg, Adelaide Maria de Souza Antunes

**Affiliations:** 1 Instituto Nacional da Propriedade Industrial, Rio de Janeiro, Brasil.

**Keywords:** Technology Transfer, Vaccine, COVID-19, Transferência de Tecnologia, Vacina, COVID-19, Transferencia de Tecnología, Vacuna, COVID-19

## Abstract

A inovação é um elemento fundamental para o desenvolvimento e crescimento, mas constituída por um processo demorado de acúmulo de conhecimento. Uma das formas de acelerar tal processo é por meio da transferência de tecnologia. Este artigo mapeou as particularidades da transferência de tecnologia para a vacina contra COVID-19, celebrado entre a AstraZeneca e o Instituto de Tecnologia em Imunobiológicos (Bio-Manguinhos), Fundação Oswaldo Cruz, bem como reconheceu os seus facilitadores, seus entraves e suas lacunas. Para tanto, foi realizada uma análise desde a etapa da seleção do parceiro mais adequado até a incorporação da nova tecnologia. A metodologia utilizada se baseou em uma ampla revisão bibliográfica sobre o tema, aliada ao estudo de caso. Os resultados apontaram que, apesar de muitas ações ainda precisarem ser realizadas para que os ganhos de capacidade tecnológica sejam potencializados, as lições aprendidas com o processo de transferência de tecnologia servirão de aprendizado e serão utilizadas nos acordos futuros e em andamento.

## Abstract (en)

Innovation is an essential element for development and growth, but it consists of a long process of knowledge accumulation, so technology transfer is used to accelerate this process. This study mapped the particularities of the technology transfer process for the COVID-19 vaccine between AstraZeneca and the Institute of Technology in Immunobiologicals (Bio-Manguinhos), Oswaldo Cruz Foundation, and identified enablers, obstacles, and gaps. Our analysis investigated the process from selection of the most suitable partner to incorporation of the new technology based on a comprehensive literature review on this topic, combined with a case study. The results showed that, although many actions still have to be performed to maximize technology capacity gains, the lessons learned from the technology transfer process will be used in future and ongoing agreements.

## Resumen

La innovación es un elemento fundamental para el desarrollo y el crecimiento, pero consiste en un proceso de acumulación de conocimiento que requiere mucho tiempo. Una de las formas de acelerar este proceso es mediante la transferencia de tecnología. Este artículo mapeó las particularidades del proceso de transferencia de tecnología para la vacuna contra la COVID-19, celebrado entre AstraZeneca y el Instituto de Tecnología en Inmunobiológicos (Bio-Manguinhos), Fundación Oswaldo Cruz, además de reconocer los facilitadores, obstáculos y brechas. Para ello se realizó un análisis, desde la etapa de selección del socio más adecuado hasta la incorporación de la nueva tecnología. La metodología utilizada se basó en una amplia revisión bibliográfica sobre el tema, combinada con el estudio de caso. Los resultados mostraron que, si bien aún es necesario llevar a cabo muchas acciones para maximizar las ganancias de capacidad tecnológica, las lecciones aprendidas del proceso de transferencia de tecnología servirán como lecciones y se utilizarán en acuerdos futuros y en curso.

## Introdução

O setor de vacina é movido pela inovação, sendo ela fundamental para se garantir uma maior vantagem competitiva. A oferta de produtos novos ou melhores constitui um dos principais elementos para o alcance do sucesso de uma empresa produtora de vacina, sendo um valor institucionalizado em sua política interna. No entanto, o processo de inovação não é algo simples, mas sim calcado em incertezas, tanto que apenas uma fração mínima é convertida em sucesso tecnológico e comercial, dada a alta complexidade do desenvolvimento de um imunizante.

Sobre esse ponto, convém mencionar que o desenvolvimento de um imunizante pode levar décadas para ser aprovado e licenciado, por ser composto por diversas fases interligadas que exigem uma extensa atividade de pesquisa e desenvolvimento (P&D), acrescida de vultosos investimentos em infraestrutura e em recursos humanos e de extensas exigências regulatórias, tanto nacionais quanto internacionais ^1^.

Desse modo, a introdução de um novo ou melhorado produto no setor de vacinas passou a ser fruto de um pequeno número de multinacionais que detêm o poder de mercado. Diante dessa alta complexidade, da necessidade de altos investimentos em projetos incertos e de longo tempo de duração, e da importância de incremento de capacitação tecnológica, os países em desenvolvimento (a exemplo do Brasil) passaram a usufruir da atividade de transferência de tecnologia, pois o domínio do processo produtivo representa uma das primeiras etapas para a constituição de capacidade inovadora ^2^. Assim, a obtenção de conhecimentos externos é uma estratégia comum na indústria de vacinas dos países em desenvolvimento.

Nesse cenário, cabe destacar a ocorrência de vários acordos de transferência de tecnologia realizados no Brasil com apoio do Ministério da Saúde, que estimularam a produção nacional de vacinas e, consequentemente, possibilitaram o atendimento às demandas solicitadas pelo Programa Nacional de Imunização (PNI). Tais acordos permitiram um salto da qualidade no setor, bem como a modernização da produção nacional ^3^.

Com base nessas constatações e visando contribuir com a essa temática, este artigo tem como enfoque a análise do complexo processo de transferência de tecnologia para a vacina da COVID-19, celebrado entre a AstraZeneca e a unidade de produção Instituto de Tecnologia em Imunobiológicos (Bio-Manguinhos), Fundação Oswaldo Cruz (Fiocruz), em 2020. Uma análise dessa parceria é de extrema importância, na medida em que possibilitou, em tempo recorde, a disponibilização de um imunizante para o enfrentamento da emergência de saúde pública provocada pela COVID-19, bem como propiciou a incorporação de novas tecnologias.

## Metodologia

Trata-se de uma revisão da literatura sobre transferência de tecnologia e uma análise do estudo de caso. Em relação à revisão da literatura, foram apuradas algumas palavras-chaves, tais como “Transferência de Tecnologia”, “Inovação” e “Absorção de Conhecimento”, a partir da leitura dos textos mais importantes, e em seguida foi realizada uma pesquisa bibliográfica nas principais bases de periódicos, como o Portal de Periódicos da Coordenação de Aperfeiçoamento de Pessoal de Nível Superior (CAPES), o Google Scholar, o SciELO e o Scopus.

Em relação ao estudo de caso, destaca-se que se refere ao contrato específico firmado entre Bio-Manguinhos/Fiocruz e AstraZeneca para a produção de vacina contra a COVID-19.

Em complemento ao material bibliográfico e à análise dos documentos do contrato, realizou-se uma pesquisa de campo dentro de Bio-Manguinhos, por meio de uma série de entrevistas feitas entre abril e maio de 2022. Os colaboradores selecionados estavam relacionados diretamente com a transferência de tecnologia para produção da vacina contra COVID-19. Ao todo, foram realizadas 11 entrevistas na forma de videoconferência, com três colaboradores da alta gerência, dois gerentes de projeto, dois funcionários responsáveis pela produção da vacina, dois profissionais do Núcleo de Inovação Tecnológica (NIT) de Bio-Manguinhos e dois colaboradores do grupo de prospecção de Bio-Manguinhos. Após a realização das entrevistas, os resultados foram compilados por meio da análise de conteúdo.

## Resultado e discussões da transferência de tecnologia em Bio-Manguinhos

### Transferência de tecnologia

A expressão “transferência de tecnologia”, de acordo com os autores Takahashi & Sacomano ^4^ (p. 189), deve ser reconhecida como “*um processo complexo que engloba a identificação da tecnologia a ser transferida, a seleção dos modos e dos mecanismos de transferência e a completa implementação e absorção da tecnologia*”.

Em complemento, Gadelha ^5^ (p. 39) menciona que a transferência de tecnologia consiste em uma “*ferramenta para diminuição do gap tecnológico, e também em uma aposta de alto risco*”. Isso porque, “*caso não haja um grande esforço para o desenvolvimento tecnológico, é possível que, quando o ciclo da tecnologia tiver sido completamente transferido, a fronteira do conhecimento já tenha se deslocado*”.

Como se vê, os autores citados a relacionam ao deslocamento do conhecimento do fornecedor para a firma receptora, bem como destacam a sua complexidade. Há, portanto, o movimento do conhecimento tácito e codificado, que poderá ou não ser apropriado a depender da competência técnica e organizacional da instituição receptora.

Após descrever brevemente algumas definições, convém discorrer sobre as principais etapas do processo de transferência de tecnologia ^6^. A atividade inicial para o alcance do sucesso de uma transferência de tecnologia consiste na identificação do problema ou da melhoria que se pretende alcançar com a busca da solução tecnológica. Em sequência, devem ser levantadas as instituições que possam ofertar a solução almejada, para que assim se realize o estudo de viabilidade. Tal estudo objetiva aferir a exequibilidade e a possibilidade de sucesso. É nessa fase que ocorre a análise das diversas opções tecnológicas até que seja selecionada aquela que mais se adequa aos objetivos estratégicos da firma recipiente. Além disso, também é verificado o nível de maturidade tecnológica, a disponibilidade de mão de obra, os equipamentos e outros elementos, para aferir se a instituição receptora apresentará capacidade de absorver a nova tecnologia e adaptá-la.

Depois de ser selecionada a instituição mais adequada para ofertar a solução tecnológica, realiza-se a fase de negociação e, em seguida, deve haver a celebração do contrato. Em relação à redação do contrato, destaca-se a sua dificuldade, o que justifica a necessidade de ele ser elaborado por uma equipe multidisciplinar e experiente, para evitar as lacunas e ambiguidades, reduzindo os custos de eventuais litígios e atendendo os interesses individuais das partes.

Uma vez firmado o contrato, ocorre a fase da transferência de tecnologia propriamente dita. Ao terminar tal etapa, espera-se que a tecnologia tenha sido adaptada e internalizada e que a organização receptora seja capaz de gerar novas capacidades tecnológicas ^2^.

No entanto, alguns elementos facilitadores ou intervenientes podem ocorrer em todas essas etapas, influenciando o resultado. Por isso, é fundamental compreender quais são esses elementos, na medida em que a atividade de potencialização ou exclusão é importante para o alcance do êxito de uma transferência de tecnologia.

Sobre esse aspecto, Greiner & Franza ^7^ identificaram que as principais barreiras são geradas pela nova tecnologia, pelos atores envolvidos e pelas exigências impostas pelos órgãos governamentais, classificadas em: (i) técnicas; (ii) regulatórias; e (iii) humanas.

As dificuldades técnicas ocorrem, em geral, quando há a transferência de uma tecnologia mais avançada ou quando ela ainda não foi utilizada pela firma receptora. Por essa razão, existe a necessidade do receptor ter capacidade tecnológica suficiente para conseguir manipular a tecnologia obtida, bem como assimilá-la. Assim, quanto mais complexa for a tecnologia, maior deverá ser o treinamento concedido aos funcionários, as adaptações estruturais, o investimento em P&D e a comunicação entre as partes ^4^.

As barreiras regulatórias, por sua vez, se dão pela morosidade dos órgãos burocráticos, pelas constantes mudanças normativas, pela dificuldade de compreensão da legislação e pelas inúmeras exigências sanitárias.

Os entraves voltados à área de recursos humanos se relacionam à ausência de profissionais capacitados em absorver a tecnologia, acrescida da falta de interesse em obtê-las em virtude de pouca motivação, insuficientes programas de treinamento e falta de autonomia para o desenvolvimento de projetos próprios ^8^.

Além disso, também podem surgir empecilhos a partir da inexistência de uma infraestrutura adequada para atender as fases de desenvolvimento do produto (barreira espacial) ^2^ ou quando ocorrer um baixo retorno, seja econômico, social, operacional ou de conhecimento, comparado ao investimento realizado.

Registra-se, ainda, a existência de barreira econômico-financeira, que está associada ao baixo investimento em treinamento, capacitação de pessoal, infraestrutura e manutenção de equipamentos, e ao constante corte de orçamento destinado à pesquisa ^3^.

Outros fatores que frequentemente afetam a transferência são: experiência prévia e diferença cultural. Em relação à diferença cultural, destaca-se que essa é considerada como uma das grandes barreiras de comunicação da tecnologia.

No que tange aos elementos facilitadores, além da boa relação entre o receptor e desenvolvedor e forte liderança em ambas as organizações, Kaippert ^2^ (p. 74) menciona que “*o capital humano é um elemento essencial, na medida em que é necessária uma mão de obra qualificada para absorver a tecnologia e posteriormente replicá-la*”.

Constata-se, de um modo geral, que várias atividades são recomendadas para potencializar o sucesso de uma transferência de tecnologia, razão pela qual os elementos facilitadores devem ser conhecidos e utilizados e as potenciais barreiras devem ser mitigadas.

### Transferência de tecnologia para a vacina contra COVID-19

O Ministério da Saúde levou em consideração que a Fiocruz é uma instituição de excelência na área de produção de imunizantes da América Latina e a maior fornecedora de vacinas do PNI, a designando para incorporar a tecnologia de produção da vacina contra a COVID-19 ^9^. Assim, a Fiocruz elaborou um documento com os principais elementos para a contratação da AstraZeneca, fruto do trabalho de prospecção.

Por meio da justificativa jurídica e técnica ^10^, a instituição demonstrou que a vacina candidata, desenvolvida pela Universidade de Oxford (Reino Unido) e licenciada pela empresa AstraZeneca, seria a mais adequada. A tomada de decisão foi baseada em uma análise técnico-científica voltada aos seguintes aspectos: plataforma tecnológica; fase de desenvolvimento; disponibilidade de estudos publicados e compartilhamento de dados adicionais; realização de fase clínica no Brasil; interesse em realizar a transferência de tecnologia; apresentação de proposta de negociação com o Ministério da Saúde; e introdução da vacina em iniciativas internacionais de acesso ao imunizante.

Como se vê, antes de ter firmada a parceria com a AstraZeneca foram trocadas informações com outras instituições e multinacionais, a fim de obter maiores detalhes sobre os diferentes imunizantes. Ao todo, a instituição brasileira dialogou com 19 empresas, realizou oito acordos de confidencialidade e avançou em discussões com três laboratórios, conforme consta na Ata nº 40, de 21 de outubro de 2020, do Tribunal de Contas da União ^11^.

Após a escolha da empresa, em 31 de julho de 2020, a Fiocruz e a AstraZeneca assinaram o *Memorando de Entendimento*, estabelecendo as condições gerais norteadoras do processo de negociação e fixando as diretrizes da parceria. O documento estruturou a relação entre as partes em dois instrumentos distintos: contrato de encomenda tecnológica (ETEC) e contrato de transferência de tecnologia de produção do insumo farmacêutico ativo (IFA) da vacina contra COVID-19.

Em 8 de setembro de 2020, foi firmado o contrato de ETEC com a AstraZeneca ^12^ para o desenvolvimento da vacina contra COVID-19. Tal contrato possibilitou a obtenção das 100,4 milhões de doses do IFA para processamento final da vacina, bem como as bases para a transferência de tecnologia. Com a obtenção do IFA importado, Bio-Manguinhos realizou o processamento final da vacina e, a partir de março de 2021, passou a disponibilizar o imunizante ao Ministério da Saúde ^13^.

Em 1^o^ de junho de 2021, foi assinado o contrato de transferência de tecnologia da vacina COVID-19 com a AstraZeneca ^14^. O contrato de transferência de tecnologia foi desenvolvido com base no modelo do contrato da Câmara Técnica de Ciência, Tecnologia & Inovação da Advocacia Geral da União, sendo composto por 15 cláusulas. Por sua vez, em 14 de fevereiro de 2022 foi liberado o primeiro lote de vacinas nacionais pelo controle de qualidade interno de Bio-Manguinhos ^9^. Em sequência, em 22 de fevereiro, a Bio-Manguinhos disponibilizou ao Ministério da Saúde 550 mil doses do imunizante contra COVID-19 produzidas com o IFA completamente nacional, sendo esse um dos processos de incorporação de tecnologia mais rápidos da Unidade. Ao todo, o Ministério da Saúde contratou 105 milhões de doses para o ano de 2022, sendo que 45 milhões foram da vacina produzida totalmente no Brasil e as demais foram com o IFA importado ^15^.

### Análise da transferência de tecnologia em Bio-Manguinhos

Por meio da pesquisa de campo foram apurados os principais aspectos que viabilizaram o sucesso da transferência de tecnologia entre Bio-Manguinhos/Fiocruz e AstraZeneca, bem como as barreiras encontradas durante o processo de incorporação e algumas das medidas empregadas para solucioná-las. Os resultados serão descritos nas seguintes fases: (i) prospecção tecnológica; (ii) negociação e formalização dos instrumentos contratuais; e (iii) produção de vacina.

#### Prospecção tecnológica em Bio-Manguinhos

A atividade de prospecção começou a ser estruturada em outubro de 2019, com a finalidade de ampliar a inteligência competitiva de Bio-Manguinhos. No entanto, com a pandemia, em janeiro de 2020 tal atividade foi redirecionada ao mapeamento das iniciativas em andamento no Brasil e no mundo relacionadas à COVID-19.

A rede de prospecção realizou um trabalho de pesquisas, levantamento e tratamento das informações obtidas em bases de dados. De início, foi feito o monitoramento da evolução do desenvolvimento de novos produtos. Os dados foram acessados em diferentes canais, envolvendo desde as fontes primárias (contatos institucionais e comunicados de imprensa) até as secundárias (como AdisInsight - https://adisinsight.springer.com/, Clinical Trials.Gov - https://clinicaltrials.gov/, e International Clinical Trials Registry Platform - https://www.who.int/clinical-trials-registry-platform/about). Também foi utilizada a base de dados privada Questel Orbit (https://www.questel.com/) para identificar as patentes.

Tais dados foram complementados com as informações extraídas dos relatórios da Organização Mundial da Saúde (OMS) e de outras instituições, *sites* especializados e páginas de laboratórios farmacêuticos. Após o levantamento, ocorreu a exclusão das informações repetidas, incompletas ou imprecisas e a sua categorização, a fim de facilitar a visualização. Depois desse trabalho, levantou-se os projetos para as vacinas contra COVID-19 em diferentes etapas. Em seguida, foram feitas reuniões com os desenvolvedores dos projetos mais avançados e aderentes às competências institucionais. As discussões com os potenciais parceiros foram importantes, já que muitas informações não tinham sido divulgadas publicamente. A decisão do governo depois da realização das análises prospectivas de Bio-Manguinhos sobre os aspectos tecnológicos, científicos, econômicos e clínicos das diferentes vacinas em desenvolvimento foi pela celebração da ETEC com a AstraZeneca.

Observa-se que a tarefa do grupo de prospecção foi complexa e dificultosa, a medida em que foi necessário o preenchimento de lacunas de conhecimento por envolver uma doença nova e uma tecnologia de ponta, assim como foi preciso um trabalho contínuo de monitoramento de dados para corrigir e atualizar as informações, tendo em vista que a atividade prospectiva estava sendo realizada em conjunto com o desenvolvimento dos imunizantes candidatos. Acrescida à dificuldade de acompanhar o desenvolvimento, também houve a questão da incerteza. Muitas plataformas tecnológicas ainda não tinham sido utilizadas, o que dificultava a previsão sobre a probabilidade de sucesso, do mesmo modo que havia o risco quanto à produção em larga escala e armazenamento.

Assim, as principais dificuldades foram: incerteza; informação limitada e imperfeita; assimetria de informações e barreiras ao acesso dos dados; elevado volume de informações; infodemia e *fake news*; desconhecimento inicial sobre o vírus, urgência de saúde pública; e dificuldade de interação diante do trabalho remoto.

Apesar da existência de vários desafios, a rede de prospecção foi um dos fatores que contribuiu para o sucesso da transferência de tecnologia, sendo um dos ganhos alcançados. A atividade prospectiva possibilitou compreender o problema e o produto que se pretendia alcançar, assim como permitiu o levantamento das instituições parceiras capazes de ofertar a solução tecnológica.

#### Negociação e formalização dos instrumentos contratuais

Após a seleção da empresa AstraZeneca, foi iniciada a negociação. Internamente, a tratativa teve o envolvimento da área de negócios e da diretoria geral de Bio-Manguinhos, bem como da presidência da Fiocruz. Além da participação dessas áreas, houve a participação: da Procuradoria Federal da Fiocruz, da Coordenação de Gestão Tecnológica da Fiocruz, do Instituto de Pesquisa Econômica Aplicada, da Câmara Nacional de Pesquisa, Desenvolvimento e Inovação da Advocacia Geral da União, e da Câmara de Deputados.

Um dos pontos positivos da negociação foi o fato da AstraZeneca já ter disponibilizado várias informações técnicas antes mesmo de ser iniciada a tratativa, o que viabilizou a avaliação de aspectos científicos-tecnológicos em detalhes mais aprofundados por Bio-Manguinhos. Em maio de 2020, foi firmado o acordo de confidencialidade entre as partes. Após a sua assinatura, reuniões semanais passaram a ocorrer, possibilitando o repasse de informações e um melhor preparo da instituição. Tanto é que, na metade de junho de 2020, a AstraZeneca iniciou a transferência da metodologia analítica. No entanto, não obstante o fornecimento de dados que somente são disponibilizados com a assinatura do instrumento de transferência de tecnologia, as informações sobre o controle de qualidade do produto final e sobre as etapas de formulação e envase apenas foram divulgadas com maior especificidade após a assinatura do contrato.

Por outro lado, um dos principais obstáculos foi a incerteza. Isso porque foram adquiridas aproximadamente 100,4 milhões de doses sem haver garantia de que a vacina atingiria o seu estágio final. Apesar da incerteza sobre o desenvolvimento e manutenção da imunidade e da possibilidade dos resultados não serem entregues, o Governo Federal entendeu ser necessário o risco de pesquisa e produção perante a urgência da manutenção da saúde pública.

Além desse desafio, também se ressalta a questão temporal. Diante da situação de emergência, houve um pequeno intervalo de tempo para a negociação e formulação dos instrumentos jurídicos. Uma estratégia utilizada por Bio-Manguinhos que possibilitou a assinatura do contrato em poucos meses foi o desentranhamento do instrumento de encomenda tecnológica da transferência de tecnologia. Como a negociação da tecnologia do IFA envolvia aspectos delicados e complexos que atrasariam o início da parceria, a instituição optou por realizá-los separadamente, visando acelerar o processo. Outros fatores que contribuíram para a redução temporal foi a existência de profissionais dedicados a projetos específicos e as suas longas jornadas de trabalho. Muitas reuniões eram feitas nos finais de semana e a carga horária de trabalho dos funcionários de Bio-Manguinhos foi ampliada, tendo o trabalho em *home office* e o entusiasmo dos profissionais contribuído para tanto.

A questão linguística também foi outro ponto crítico, haja vista que o idioma adotado foi o inglês. Aliado ao idioma estrangeiro, as diferenças culturais também dificultaram as tratativas e a comunicação entre as partes.

Além destes pontos, a complexidade jurídica também foi um obstáculo, dada a dificuldade do desenho jurídico do contrato. Ao mesmo tempo em que precisava ser redigido um instrumento de ETEC, até então nunca feito pela instituição brasileira, também era necessário incorporar nesse documento a transferência de tecnologia, assim como era preciso respeitar as condições precedentes, diante da existência de uma licença principal da Universidade de Oxford para a AstraZeneca, e evitar o uso de cláusulas restritivas.

Outra questão foi a dificuldade da AstraZeneca em entender a legislação brasileira sobre a encomenda tecnológica. O acordo com a Fiocruz era diferente de todas as outras parcerias celebradas pela AstraZeneca, já que não envolvia uma compra pura e simples, mas um projeto de pesquisa e desenvolvimento ainda não terminado. Tal ponto foi de enorme estranheza por não fazer parte do modelo de negócio da empresa. Para o melhor entendimento da AstraZeneca sobre o objeto contratual e para que fosse aceita a inserção no contrato da encomenda de um projeto em desenvolvimento tecnológico, foi necessária a realização de várias reuniões.

A empresa também teve dificuldade em compreender os limites de uma instituição governamental, havendo resistência em aceitar que a Fiocruz tem seu funcionamento regulado por normas de direito público por ser uma fundação pública. Também houve dificuldade em ser entendida a parte burocrática e a quantidade de instâncias necessárias para aprovação do contrato.

Acrescida a essas adversidades, houve o desafio em realizar as etapas prévias à celebração da ETEC de forma acelerada. Diante da necessidade de encurtar as etapas e com base na Lei de Emergência Nacional, criou-se um paralelismo entre as fases.

Todo o processo de formalização do contrato e as suas etapas prévias foram bastante complexas. Apesar de os profissionais de Bio-Manguinhos possuírem familiaridade com os acordos de transferências e da instituição apresentar uma equipe multidisciplinar, houve uma enorme dificuldade durante a negociação e elaboração das cláusulas contratuais.

#### Produção da vacina contra COVID-19 em Bio-Manguinhos

#### (a) Dificuldades na fase de produção

Em adição aos desafios existentes na fase de negociação, também houve barreiras durante a fase de produção. Novamente, a questão temporal foi um elemento problemático. Diante da urgência em ser implementada a produção do imunizante contra COVID-19, houve um espaço de tempo extremamente curto para serem realizadas as adaptações fabris e treinamentos. No entanto, apesar da questão temporal ser vista como um empecilho ela também foi enquadrada como um ponto positivo. Isso porque um dos problemas comuns nos processos de transferência de tecnologia, celebrados por Bio-Manguinhos, é o seu grande tempo de duração. Muitas vezes quando uma tecnologia vem a ser incorporada pela instituição ela já está obsoleta, dada a longa demora para a conclusão do processo de transferência. Nesse caso em específico, a disposição de vontade da AstraZeneca, a existência de uma base tecnológica em Bio-Manguinhos e o aporte financeiro foram alguns dos fatores que contribuíram para que a tecnologia fosse transferida rapidamente.

Noutro rumo, a aquisição de insumos e equipamentos em um mercado sob grande demanda também foi um forte empecilho. Em relação à escassez deles, Bio-Manguinhos adotou as seguintes ações: intensivo acompanhamento; frequentes contatos com os fornecedores para garantir que os insumos fossem entregues nas datas previstas; e desenvolvimento de soluções alternativas.

Entre essas soluções, destaca-se a inicialização do processamento final com a substituição de alguns aparelhos pelos que já existiam na instituição brasileira e o ajuste do produto ao processo já estabelecido em Bio-Manguinhos. Tal adaptação somente foi possível dada a experiência de Bio-Manguinhos, acrescida da articulação com a AstraZeneca. O procedimento de ajuste feito por essa unidade de produção foi bem aceito pela empresa estrangeira. Inclusive, os outros parceiros da AstraZeneca trabalharam dessa mesma forma, dada a dificuldade de compra dos suprimentos.

Deve ser observado que a adequação do produto ao processo de Bio-Manguinhos foi uma estratégia inovadora, já que as transferências realizadas anteriormente pela instituição seguiam exatamente o procedimento adotado pela desenvolvedora, o que por um lado diminui o risco tecnológico, e por outro aumenta consideravelmente o tempo. Nota-se que o procedimento de ajuste foi uma estratégia de aceleração, pois se houvesse a necessidade de aguardar a compra de todos os equipamentos, seria despendido um tempo consideravelmente maior.

Além das dificuldades mencionadas, a capacidade de processamento final durante a fase inicial também foi uma questão problemática. Por um lado, Bio-Manguinhos precisava buscar respostas e apoiar o Ministério da Saúde no enfrentamento, mas por outro, precisava respeitar o cronograma de fornecimento de outros produtos ao governo. Diante disso, foi necessário revisar todo o planejamento e o controle das operações, avaliar as prioridades para manter as atividades essenciais, formular uma nova lógica de trabalho em regime remoto e montar novas estruturas de governança e gestão. A realização desse replanejamento possibilitou a ampliação da capacidade de processamento final.

Com a superação da dificuldade de capacidade de processamento final, o desafio que passou a prevalecer foi o da falta do IFA e do seu não recebimento nas datas acordadas por problemas burocráticos. Para tanto, foram realizadas reuniões com a AstraZeneca e foi obtido apoio do Ministério das Relações Exteriores para a realização de contatos com a Embaixada da China, local de origem dos insumos.

De igual modo, a falta de pessoal também foi um grande problema, já que uma média de 40% da mão de obra estava afastada para trabalhar em regime de *home office*. Como forma de remediar a situação, foi criado um canal de comunicação entre os gestores e os colaboradores, bem como ocorreu a contratação de novos profissionais. Houve a necessidade de treinamento aos novos funcionários.

Por outro lado, a terceirização também deve ser considerada um dos problemas gerais de Bio-Manguinhos, já que a instituição realiza essa forma de contratação a fim de remediar a escassez de mão de obra, tendo em vista que o número de concurso público não acompanha as necessidades da organização. Sobre esse ponto, vale relembrar que a quantidade de profissionais permanentes vem diminuindo, progressivamente, no decorrer dos anos. É notório que tal fato gerou um enorme prejuízo à administração pública, já que muitos dos profissionais terceirizados estão à frente de projetos, e o quadro permanente de servidores efetivos no ano de 2020 representava apenas 10,91% do total ^16^.

Ainda sobre a terceirização, salienta-se a alta rotatividade desses profissionais diante da inexistência de vínculo permanente com a instituição. É inequívoco que a alta rotatividade gera uma perda de investimento e conhecimento para a administração pública, sendo imprescindível que a instituição assegure a formação e a retenção de profissionais altamente especializados.

Somado a esses desafios, convém registrar a existência de dificuldades operacionais. Entre essas se destacam as seguintes: reorganização da produção e logística; preparação das instalações para a produção da vacina contra COVID-19; contratação de pessoal; treinamento profissional; e instauração de um regime de produção sem folga.

Também vale salientar a questão da dependência de liberação orçamentária de Bio-Manguinhos como um dos grandes problemas enfrentados. Nos últimos anos, ocorreu uma redução dos gastos em pesquisa, provocado pelo corte no orçamento federal à saúde. Nesse sentido, registra-se que, em 2019, apenas R$ 70 milhões foram gastos em P&D, o que corresponde a 3,2% da receita total, sendo esse investimento similar ao do ano anterior ^17^. A baixa taxa de investimento em P&D em relação à receita total da instituição, em comparação com as multinacionais farmacêuticas, contribui para o cenário de dependência. Isso porque para manter a qualidade, é necessário que o investimento seja contínuo.

Em que pese a baixa destinação orçamentária dos últimos anos, ocorreu uma ampliação dos investimentos em virtude da pandemia. Em 2020, Bio-Manguinhos investiu aproximadamente R$ 140 milhões em P&D ^16^, o que corresponde ao dobro do valor investido no ano anterior. No entanto, o valor investido equivale a 2,57% da receita total, que passou de R$ 2,18 bilhões para R$ 5,29 bilhões.

É notório que o investimento deve ser constante, não podendo ser esporádico. Como demonstrado, é fundamental que Bio-Manguinhos realize continuamente o desenvolvimento de suas equipes, assim como modernize e amplie a sua infraestrutura para que sejam alcançados altos níveis de eficiência e, consequentemente, seja aumentada a sua capacidade produtiva e tecnológica.

Desse modo, há a necessidade de priorizar e impulsionar mais recursos à ciência, tecnologia, pesquisa e inovação, a fim de fortalecer as instituições públicas e ampliar a capacidade humana.

#### (b) Elementos facilitadores na fase da produção

Apesar da presença desses entraves, vários elementos foram facilitadores. O primeiro a ser mencionado é a credibilidade da instituição e de seu corpo de pesquisadores. Em complemento, destaca-se a capacidade da instituição. Apesar da plataforma tecnológica de vacina contra COVID-19 da AstraZeneca/Oxford ser inovadora, ela apresentava uma similitude com as competências de Bio-Manguinhos. Além dessas semelhanças, muitos dos conhecimentos já estavam incorporados dentro dessa unidade de produção da Fiocruz, o que facilitou a manipulação da nova tecnologia e a sua assimilação. Outro elemento foi o fato de Bio-Manguinhos já ter celebrado várias parcerias. Tais experiências, além de terem permitido a incorporação dos conhecimentos, também possibilitaram o aprimoramento do processo e a não repetição de erros.

A transparência e a comunicação efetiva com a AstraZeneca também devem ser considerados como pontos positivos. Por ser uma questão pandêmica, a comunicação foi bastante facilitada e a negociação foi estendida para a obtenção de maiores ganhos. Uma vez que a AstraZeneca estava empenhada em produzir a vacina e introduzi-la ao mercado e Bio-Manguinhos necessitava incorporar a tecnologia, por motivos de saúde pública. Dentro da instituição brasileira, houve o compartilhamento de informações entre os diferentes departamentos e equipes.

Além da boa comunicação entre as partes envolvidas, observou-se a motivação pessoal dos colaboradores de Bio-Manguinhos e a presença de profissionais capacitados. Também se visualizou uma vontade em transmitir a tecnologia por parte da fornecedora, sendo realizados treinamentos e inúmeras reuniões, as quais eram feitas, no mínimo, uma vez por semana, sendo tratadas as dúvidas e a maneira como Bio-Manguinhos precisava se estruturar para absorver a tecnologia.

Quanto aos treinamentos, destaca-se que sempre eram fornecidos antes de ser inicializada alguma etapa do processo. Tais treinamentos ocorreram de forma remota diante da pandemia e do curto espaço de tempo, ao contrário dos processos tradicionais de transferência. O treinamento remoto foi, ao mesmo tempo, uma oportunidade e uma dificuldade para as partes envolvidas. Por um lado, um maior número de pessoas pôde participar do treinamento, não havendo a necessidade de compartilhamento do conhecimento interno e tampouco esteve presente o problema de os terceirizados não poderem realizar viagens internacionais. Por outro lado, a absorção do conhecimento foi um pouco prejudicada.

Além dos treinamentos e das reuniões, também houve o auxílio presencial de dois consultores contratados pela AstraZeneca, que ficaram aproximadamente quatro meses em Bio-Manguinhos prestando suportes na implementação do processo. Após os consultores visualizarem que a instituição tinha domínio, eles deixaram de comparecer presencialmente; porém, continuaram participando das reuniões técnicas.

Outro elemento fundamental foi a realização, por parte de Bio-Manguinhos, de uma análise aprofundada sobre a nova tecnologia e sobre a sua compatibilidade com a existente dentro da instituição. As informações obtidas durante a fase de prospecção subsidiaram a negociação e a elaboração dos contratos, tendo sido fundamental o conhecimento detalhado do grau de maturidade da tecnologia.

A utilização de uma infraestrutura já existente também contribuiu para a redução do tempo e dos custos. Durante a incorporação do IFA, como mencionado anteriormente, ocorreu a adaptação do processo, sendo utilizada a estrutura existente em Bio-Manguinhos para o processamento final. Por sua vez, para a produção do IFA, houve uma adequação maior do Centro Henrique Penna, ocorrendo o aproveitamento de vários instrumentos e a obtenção de equipamentos específicos, como biorreatores e sistemas de purificação. Também foi necessário construir um novo laboratório para suprir o aumento da demanda por análises de controle de qualidade, assim como foi preciso construir uma nova área para armazenar o IFA.

A organização do trabalho em Bio-Manguinhos também contribuiu bastante para o sucesso, tendo sido montada uma estrutura de gestão robusta. A instituição estabeleceu uma gerência de projeto, constituída por aproximadamente 30 funcionários e por um gerente geral que respondia diretamente ao diretor de Bio-Manguinhos, garantindo maior rapidez na tomada de decisões e na solução dos problemas. O gerente geral tinha quatro frentes de trabalho: (i) gerência administrativa (*compliance* legal, aquisições, cadeia de suprimento, adequações de quadro pessoal, treinamento etc.); (ii) gerência de transferência de tecnologia propriamente dita (processamento final, produção de ifa, controle de qualidade); (iii) gerência de infraestrutura; e (iv) *compliance* regulatório e recursos humanos (atendimento aos requisitos regulatórios: biossegurança, segurança do meio ambiente, segurança do trabalho, boas práticas de fabricação).

Por fim, destaca-se o papel fundamental da iniciativa privada. Além de auxiliar na negociação com os fornecedores de insumos e equipamentos, também contribuiu financeiramente. A colaboração da Companhia de Bebidas das Américas (AMBEV) foi primordial, cedendo dez funcionários para ajudarem com o processo da transferência de tecnologia.

Sobre as doações, registra-se que até maio de 2022 a Fiocruz, em parceria com indivíduos e organizações públicas e privadas, recebeu a quantia de aproximadamente R$ 505 milhões. Parte desses recursos foi utilizada para melhoria da infraestrutura e adaptação fabril ^18^. O aludido aporte também contribuiu para que Bio-Manguinhos inaugurasse, em 23 de novembro de 2021, o Laboratório Físico-químico (LAFIQ), a fim de suprir o aumento da demanda por análises de controle de qualidade provocado pela incorporação da produção da vacina contra o novo coronavírus.

Desse modo, o apoio da iniciativa privada, a experiência na área de biotecnologia, a infraestrutura tecnológica instalada e a capacidade de gestão foram fundamentais para a incorporação da tecnologia de forma rápida.

## Considerações finais

A pandemia provocada pelo SARS-CoV-2, além de ter mostrado o quanto é incipiente a indústria biotecnológica brasileira e o quão frágeis são as políticas públicas que estimulam as atividades de P&D, reafirmou a importância das instituições governamentais e a relevância da autonomia local e regional na produção de imunizantes e insumos estratégicos para a saúde pública nacional e internacional. A dependência de insumos e equipamentos torna o sistema de saúde completamente vulnerável.

No entanto, nos últimos anos, o cenário brasileiro de pesquisa, desenvolvimento tecnológico e inovação em relação aos imunizantes tem sido de retrocesso, diante da diminuição de recursos financeiros e das escassas políticas públicas que estimulam as atividades de P&D.

Apesar de serem poucas as iniciativas e ínfimos os investimentos governamentais, Bio-Manguinhos vem celebrando parcerias para absorção de novas tecnologias, visando superar a situação de dependência nacional, ampliar o conhecimento e atender as demandas do PNI. O esforço por parte dessa unidade de produção deve ser visto não somente como um elemento estratégico para a saúde pública, mas principalmente como uma forma de fomentar a inovação e acentuar o crescimento econômico.

Por meio dos acordos de transferência de tecnologia, muitos ganhos têm sido alcançados por Bio-Manguinhos. Porém, muitos obstáculos ainda precisam ser superados. A presença reiterada de alguns entraves nas parcerias apenas reafirma a necessidade de uma política pública de longo prazo com investimentos e monitoramento frequentes, bem como a necessidade de serem ampliadas as ações formais voltadas à capacitação de pessoal e a destinação de recursos financeiros às atividades de P&D.

Por fim, ressalta-se que as lições aprendidas com o processo de transferência de tecnologia para a vacina contra a COVID-19, celebrado pela Bio-Manguinhos/Fiocruz, certamente servirão de aprendizado e serão utilizadas nos acordos futuros e em andamento. A incorporação da tecnologia e a produção de um imunizante totalmente nacional ocorreu em um tempo extremamente curto, o que realçou ainda mais a competência existente nessa instituição governamental.
